# Spatial Distribution of Cryptic Species Diversity in European Freshwater Amphipods (*Gammarus fossarum*) as Revealed by Pyrosequencing

**DOI:** 10.1371/journal.pone.0023879

**Published:** 2011-08-31

**Authors:** Anja Marie Westram, Jukka Jokela, Caroline Baumgartner, Irene Keller

**Affiliations:** 1 Aquatic Ecology, Eawag, Swiss Federal Institute of Aquatic Science and Technology, Dübendorf, Switzerland; 2 ETH-Zürich, Institute of Integrative Biology, Zürich, Switzerland; 3 Fish Ecology and Evolution, Eawag, Swiss Federal Institute of Aquatic Science and Technology, Kastanienbaum, Switzerland; Biodiversity Insitute of Ontario - University of Guelph, Canada

## Abstract

In order to understand and protect ecosystems, local gene pools need to be evaluated with respect to their uniqueness. Cryptic species present a challenge in this context because their presence, if unrecognized, may lead to serious misjudgement of the distribution of evolutionarily distinct genetic entities. In this study, we describe the current geographical distribution of cryptic species of the ecologically important stream amphipod *Gammarus fossarum* (types A, B and C). We use a novel pyrosequencing assay for molecular species identification and survey 62 populations in Switzerland, plus several populations in Germany and eastern France. In addition, we compile data from previous publications (mainly Germany). A clear transition is observed from type A in the east (Danube and Po drainages) to types B and, more rarely, C in the west (Meuse, Rhone, and four smaller French river systems). Within the Rhine drainage, the cryptic species meet in a contact zone which spans the entire *G. fossarum* distribution range from north to south. This large-scale geographical sorting indicates that types A and B persisted in separate refugia during Pleistocene glaciations. Within the contact zone, the species rarely co-occur at the same site, suggesting that ecological processes may preclude long-term coexistence. The clear phylogeographical signal observed in this study implies that, in many parts of Europe, only one of the cryptic species is present.

## Introduction

A fundamental problem for applied as well as basic biodiversity research is that we often do not know to what extent morphological and genetic diversity are correlated. Phenotypic plasticity can lead to pronounced morphological differentiation despite genetic similarity. At the other extreme, morphological stasis can completely mask genetic diversification, and molecular analyses are necessary to identify distinct lineages, subspecies or species within a morphospecies. Due to their separate evolutionary histories, such genetic groups may possess unique adaptations and evolutionary potential and, hence, may be distinct evolutionarily significant units [1] relevant for conservation. This is especially true for cryptic species, which are often reproductively isolated and therefore true species under the biological species concept [2], and often millions of years old [3], but morphologically indistinguishable.

In some taxa, these issues are well understood and are considered in protection and management programs, for example in fishes, like trout *Salmo trutta* and Atlantic salmon *Salmo salar* [4]. However, many other common and widespread taxa might exhibit similarly strong cryptic diversity (e.g. [3], [5]). As cryptic species may differ in biological characteristics, correct species identification will be fundamental to ensure the comparability between studies in basic and applied research. Knowledge on the geographical distribution of cryptic taxa will be greatly beneficial in this respect, because in case phylogeographic signals are strong, it may be possible to reliably predict the species present in a particular region without time-consuming molecular analyses.

The organism we investigate in this study is the freshwater amphipod *Gammarus fossarum* Koch (Crustacea, Amphipoda). Amphipods are a central element of aquatic ecosystems, for example as fish prey [6] or shredders of organic material [7] and intermediate hosts for several fish and bird parasites of the phylum Acanthocephala [8]. *G. fossarum* is widespread in Central Europe [9], [10], especially in the upstream reaches of streams [11]. *G. fossarum* is vulnerable to human activities [12], [13] and is often used in ecotoxicological assays, or in ecological assessment as indicator of habitat quality, for example in the “Modul-Stufen-Konzept” for Switzerland [14].

Several studies indicate the presence of at least three cryptic species within *G. fossarum*. As early as 1989, Scheepmaker and van Dalfsen found large allozyme differentiation within *G. fossarum* in Europe [15], leading to the distinction between *G. fossarum* sensu stricto and *G. fossarum* sensu lato. This subdivision was refined by Müller [16], [17] using sequencing and allozyme data to describe three genetically distinct cryptic species - types A, B and C. Allozyme and mtDNA (16S rRNA gene) genotypes were concordant in mixed type A / type B populations, suggesting complete reproductive isolation between the types [17]. Large genetic differentiation indicates that the species split several million years ago [17], while morphological differentiation is not sufficient for species discrimination [18].

Müller found a geographical distribution pattern with type A in the east and type B in the west of his study area (mostly central and Southern Germany and surrounding areas), while type C was rare and only occurred in western populations [17]. However, as *G. fossarum* prefers upstream reaches of streams [11], it tends to be rarer than other *Gammarus* species in lowland areas like the Netherlands and many regions of Germany [13], [19], [20], [21]. Previous studies analyzing the *G. fossarum* cryptic species distribution have therefore not focused on the areas where *G. fossarum* is the dominant *Gammarus* species, and consequently ecologically especially relevant, like Switzerland or Austria (own observations and [22]).

In this study we compile data from previous studies analyzing the cryptic species distribution and complement this data set with new samples mainly from Switzerland, applying a novel pyrosequencing assay for rapid molecular species identification. The resulting data set allows a detailed description of the current species distribution in Central Europe and insights into the historical processes which produced it.

## Results

In Switzerland, we analyzed 62 *G. fossarum* populations with regard to cryptic species composition. Complete 16S sequencing of 137 individuals from across Switzerland revealed all three cryptic species described by Müller [17]. Based on the full sequencing and pyrosequencing dataset, we detected 24 type A populations, 26 type B populations, and three type C populations (Table S1) in Switzerland. Nine populations contained both type A and type B at variable frequencies.

**Figure 1:**
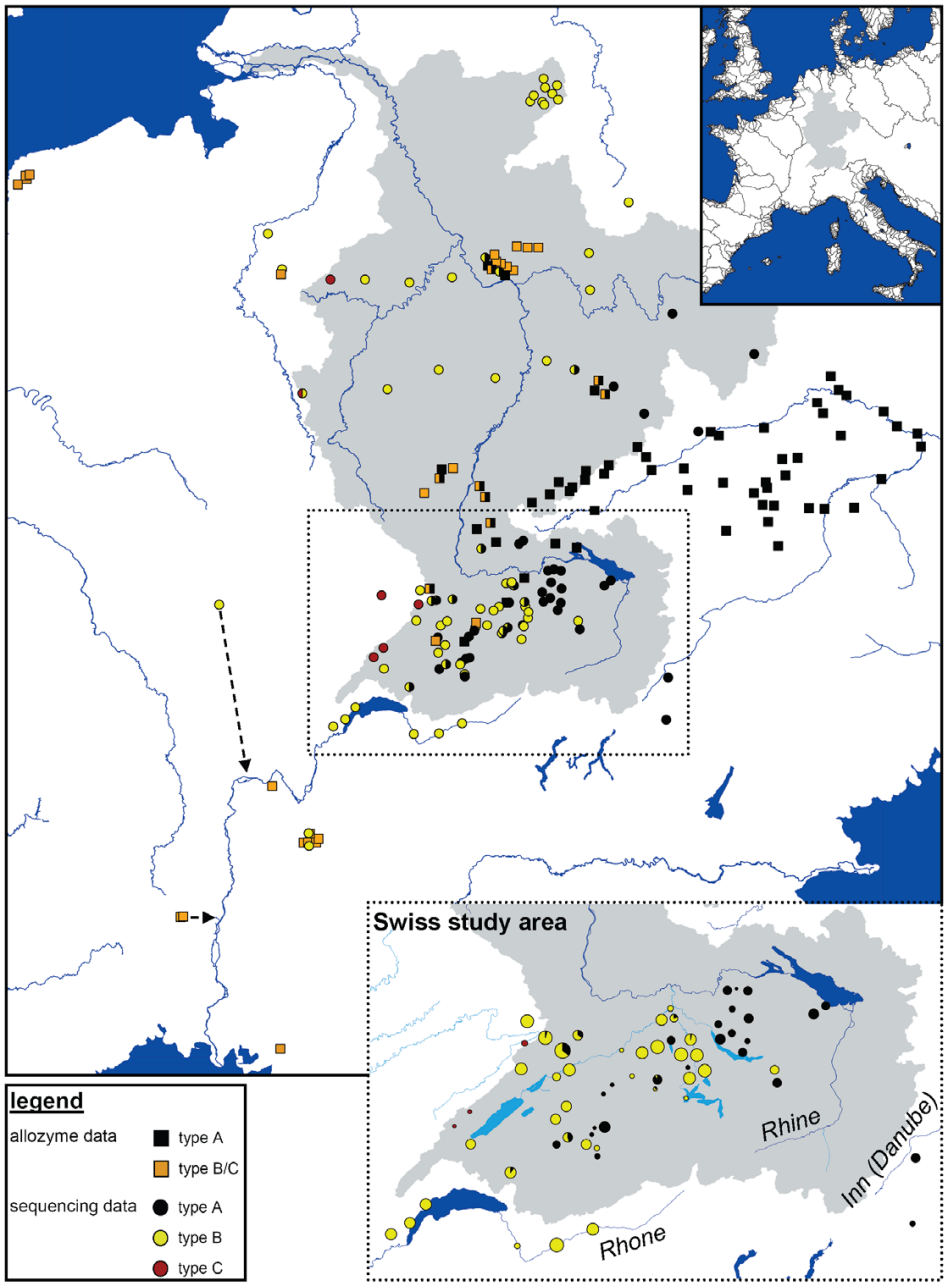
Distribution of three cryptic *Gammarus fossarum* species (types A, B and C) in Europe. The Rhine drainage, which contains the contact zone between type A and type B, is shaded in grey. The small map on the top right indicates the position of the study area within Europe. The large map contains data from six previous publications (see text) and the present study. The method used for species identification is indicated (square = allozyme analysis; circle = sequencing), as the distinction between type B and C is not possible based on allozymes. For some sites, arrows indicate the flow direction of the sampled stream. The smaller map contains only the Swiss samples collected for this study and shows the exact species compositions of the samples in pie diagrams. Pie diagram size corresponds to sample size (n between 2 and 57 per population; total n = 1337). To prevent overlap of the pie diagrams, some positions are slightly shifted. The exact positions are shown in the large map.

The distribution pattern in Switzerland (Figure 1) can be described as follows: In the east (parts of Rhine drainage, Danube drainage and Po drainage), only type A populations were found. Further west, there was an area where both type A and B occurred, occasionally within the same population. This contact zone was located within the Rhine drainage. In this area, type A was generally rarer than type B, i.e. there were fewer type A than type B populations, and within mixed populations, type A was mostly the rarer species. Four of the five Rhone populations contained type B, while type A was not observed in this drainage. Only three type C populations could be detected, which were all in the very west of Switzerland, but belonged to different drainages (Rhine and Rhone).

We compiled data from 6 studies which had analyzed 117 *G. fossarum* populations in Western and Central Europe (Figure 1; Table S2). The general pattern that emerged again was that type A dominated in the east (Danube drainage), while types B and C were more common in the west (Meuse, Rhone, Weser, and four smaller French river systems draining into the Atlantic or Mediterranean). Type C was observed in only two populations (Rhine and Meuse drainage) out of the 21 populations where a distinction between type B and C was possible. Again, all three cryptic species were observed within the Rhine drainage, and 13 populations within this contact zone contained both types A and B/C (Figure 1).

Sequencing of 64 precopula pairs (pairs formed prior to mating) from a mixed population in Glovelier revealed 10 type A and 53 type B pairs and only a single heterospecific pair consisting of a type B male and a type A female.

## Discussion

The synthesis of the data from this study and earlier publications clearly shows that, in many cases, the geographical location alone will be an accurate predictor of the cryptic *G. fossarum* species present at a particular site. Because of these mostly non-overlapping geographical distributions, the range of each cryptic species will be considerably smaller than that of the entire morphospecies. We found relatively clear distribution patterns: In Switzerland, type A is more common in the east, while type B is more frequent in the west (Figure 1). This pattern recurs on a larger scale across Central Europe: type A occurs in the eastern drainages (Danube and Po), while type B is common in the west (Meuse, Rhone, Weser, and four smaller French river systems). A long contact zone spans the Rhine drainage from central Germany down to the Alps in Switzerland, covering the whole latitudinal distribution range of the species complex at this longitude. This contact zone does not coincide with any obvious environmental clines, for example forests streams with stones as well as grassland streams with more macrophytes were available across the entire sampling region. There is also no major geological cline which could explain the differential distribution of the species. This suggests that the species distribution is heavily shaped by past recolonization processes, leading to mainly geographical instead of ecological sorting of the species.

It is currently unclear if type C is generally very rare, or if the centre of its distribution range was not covered in this survey. A small number of type C populations was observed along both sides of the Western border of the Rhine drainage. Only five samples where types B and C can be distinguished are available from further west in the Rhone and Meuse systems. Interestingly, all of these are type B, which would speak against a widespread occurrence of type C in Western Europe.

The finding that, outside the contact zone, geographical sampling location is highly predictive of species composition will be of interest in applied fields of research where correct species identification is important. Members of the *G. fossarum* cryptic species complex are regularly used in ecotoxicological studies (e.g. [23], [24]). They are sensitive to anthropogenic acidification and pollution [12], [13], which may cause local extinctions with potentially serious impacts on ecosystem functioning [13]. In such studies, species identification is normally limited to the level of the morphospecies even though the cryptic species are known to differ slightly in their ecological requirements [25], [26]. Consequently, they may also react differently to environmental change, and results or predictions obtained from one species need not hold for another.

For situations where molecular species identification is still necessary (i.e. when samples are taken from within the contact zone), pyrosequencing proved to be a rapid and cost-effective method for the large-scale identification of cryptic species from multiple populations. The assay is technically easy to implement and requires very little optimization. The efficiency of molecular species identification based on pyrosequencing could, in principle, be further increased through the analysis of bulk samples, i.e. mixtures of DNA from multiple individuals (e.g. [27]). However, preliminary tests showed that estimates of the relative frequency of types A and B in mixtures of known composition were unreliable. A possible explanation could be the large sequence divergence between the cryptic species, which could lead to unequal amplification efficiency in the PCR.

The evolutionary distinctness of the different *G. fossarum* types is underlined by the extent of reproductive isolation observed between them. Although the species were found to be partly interfertile under laboratory conditions ([28]; references therein), our small sample of precopula pairs indicates a preference for conspecific mates. This is in agreement with the results from genetic analysis, which suggested the absence of hybrids in mixed populations [17]. In the field, assortative mating could be promoted for example if the species sort into different microhabitats [26], which is not possible under lab conditions.

The observed geographical distribution of type A and types B/C is not only interesting for applied research, but also allows the inference of past recolonization processes. The cryptic *G. fossarum* species probably split well before the onset of the Pleistocene glaciations [17]. The fact that we observe a clearly separate geographic distribution of type A and types B/C indicates persistence of the species in different refugia during the Pleistocene glaciations. Müller [16] suggests that type A survived in an eastern and type B in a western refugium. The contact zone would have formed when the colonization fronts met in Central Europe. The eastern refugium was probably located in the Danube basin, where only type A occurs. The Danube basin also played a central role as a refugium for multiple fish species [29], [30]. From there, recolonization of Central Europe by type A might have taken place in a north-western direction to the west of the Danube drainage, as well as the Po and Rhine drainages. The location of the refugium of type B is more difficult to infer. One possibility is a south-western European refugium, as suggested by Müller [17], for example in France or even further south on the Iberian peninsula, one of the classical Mediterranean refugia [31]. In accordance with this idea, *G. fossarum* still occurs as far south as the Pyrenees [9] and the South of France seems to harbour high genetic diversity in the form of several endemic *Gammarus* species [32]. During the less severe glaciations in the late Pleistocene, more northern, secondary refugia may also have been available [33]. Such cryptic northern refugia have also been suggested for another freshwater crustacean *Asellus aquaticus* L. [34].

All probable recolonization patterns would have required repeated crossing of watersheds, for example of type C across the Rhine-Rhone boundary or a spread of type A out of the Danubian drainage (Müller [17] and Figure 1 of the present study). Movements across watersheds may be explained by past landscape changes which produced connections between drainages, for example the formation of post-glacial meltwater lakes (see e.g. Figure 5 in [35]), which might also explain the geographical distributions of genetic diversity in several fish species [35], [36], [37]. More recently, transport by waterfowl [38] or anthropogenic introductions (e.g.[39]) might occasionally have led to exchanges across watersheds.

Contact zones between cryptic species can complicate research and conservation efforts because the species at a given site cannot be inferred without molecular data. However, contact zones are interesting insofar as they can enhance our understanding of the coexistence of similar species, a topic that has been debated for decades [40]. Types A and B co-occur in a contact zone within the Rhine drainage that is quite narrow especially in the more northern part (Germany). Within the contact zone, we find remarkably few mixed populations where types A and B coexist (Figure 1), suggesting that interference or exploitative competition probably prevents long-term coexistence. *Gammarus* species often are strong intraguild predators [7]. If one species showed a stronger cannibalistic tendency, this could dramatically affect the probability of coexistence [41]. Additionally, or alternatively, one species could be better adapted to a particular local environment. Indeed, it has been shown that type A prefers stony habitats with leaves, while type B prefers plants and muddy substrate [26]. Such mechanisms would translate into unequal population growth rates and, ultimately, displacement of the poorer competitor. The few mixed populations we do find might have evolved ecological differentiation pronounced enough to allow coexistence [42] or they might represent transitional stages before the outcompetition of one species. It is possible that one species is currently expanding its range at the cost of the other [16]. Consistent with this scenario, type B populations do not show a pattern of isolation by distance, and a decrease in the mean number of allozyme alleles towards the contact zone in the German study area [16].

## Methods

### Ethics Statement

No specific permits were required for the described field studies. In Switzerland, France and Germany, work with *Gammarus* does not require permission and waterbodies are not private property if nothing else is indicated. Samples were not taken from streams where private property was indicated or from nature reserves. The field studies did not involve endangered or protected species.

### Sampling and morphospecies identification of *G. fossarum*

We collected gammarids from 62 sites in Swiss streams by kick-sampling all available microhabitats (e.g. stony areas and macrophytes) and stored them in 70% ethanol (Table S1). To increase the European dataset, we also added samples from several sites outside of Switzerland (Table S1). These included five French populations (Rhone drainage), one population from southern Germany (Rhine drainage; provided by Andreas Bruder, Eawag, Switzerland) and nine populations from the very north of the Rhine drainage in Germany (provided by Michael Zeidler, University of Münster, Germany).

Using a dissecting microscope, we determined whether our samples contained *Gammarus pulex* L. [43], another amphipod species which may coexist with *G. fossarum* [21], [44]. *G. pulex* were discarded from further analyses.

In one population (Glovelier), where *G. fossarum* types A and B coexisted (see Results), we repeatedly sampled precopula pairs, i.e. pairs formed prior to mating, between March and July 2009. Genetic species identification served to detect whether premating isolation prevents the formation of mixed species pairs.

### Genetic species identification

We extracted DNA from complete *G. fossarum* individuals or heads [45]. To discriminate between the cryptic species, we used pyrosequencing [46], which is faster and cheaper than conventional sequencing. First, a short fragment containing polymorphic sites suitable for species discrimination (e.g. single nucleotide polymorphisms (SNPs), short indels) is amplified using PCR. In the pyrosequencing reaction, a primer anneals close to this diagnostic position, and the successive injection of fluorescently labelled nucleotides allows the real-time determination of the sequence of the elongating strand. In contrast to other SNP genotyping methods, it is possible to genotype two nearby SNPs (in this case separated by 6 bp) in the same assay.

Pyrosequencing requires prior knowledge of complete sequences for the detection of diagnostic positions. Therefore, we performed conventional sequencing of the 16S mitochondrial gene for 137 animals from various Swiss streams. We amplified the gene using primers and PCR conditions from Müller *et al.* [17] and purified 5 µl of the product by adding 0.5 µl exonuclease I and 2.0 µl shrimp alkaline phosphatase (Fermentas), followed by incubation at 37°C for 15 min and 85°C for 15 min. The product was sequenced using the forward PCR primer and BigDye Terminator v3.1 (Applied Biosystems) cycle sequencing reagents. The cycle protocol included 5 min at 96°C followed by 25 cycles of 96°C for 10 s, 50°C for 5 s and 60°C for 4 min. The cycle sequencing product was purified (BigDye Xterminator Kit, Applied Biosystems) and sequenced on an Applied Biosystems 3130xl Genetic Analyzer. We manually edited the sequences in the program Chromas Lite 2.01 (http://www.technelysium.com.au/chromas_lite.html) and aligned them with published sequences (GenBank accession numbers: AJ269587–AJ269627; [17]), using the ClustalW algorithm in BioEdit (version 7.0.9.0; http://www.mbio.ncsu.edu/BioEdit/bioedit.html). Each sequenced individual could be unequivocally assigned to one cryptic species by eye (due to large interspecific differences compared to typically less than 1% sequence divergence within species). In total, we found 82 type A individuals (from 22 populations), 48 type B individuals (from 13 populations), and seven type C individuals (from two populations).

We selected two diagnostic SNPs (SNPs 1 and 2) which allowed the distinction between type A and the two other types (B and C). At a third SNP, the genotype of C differed from that of either A or B (Table S3). Hence, together these three positions allowed the reliable identification of all three cryptic species. We amplified two fragments of 107 bp (containing SNPs 1 and 2) and 163 bp (containing SNP 3) respectively using the following conditions: Initial denaturation for 5 min at 95°C was followed by 45 cycles of 15 s at 95°C, 30 s at 55°C, and 30 s at 72°C and a final extension of 4 min at 72°C. Primers were taken from the literature or newly designed by eye based on our sequence data (Table S3).

SNPs 1 and 2 were genotyped in 1252 individuals on a PyroMark ID (Biotage) pyrosequencing device at the ETH Genetic Diversity Center, Zürich, Switzerland, using the standard pyrosequencing protocol provided by Qiagen. We analyzed and checked the resulting pyrograms in the “SNP mode” to assign each individual to either type A or types B/C. As expected, results based on SNP 1 and 2 were always concordant.

In all populations containing types B/C, we genotyped SNP 3 in four individuals to further distinguish between the two types. If all four animals were type B, the population was classified as pure type B, as type C was assumed to be rare. It is clear that this relatively small sample size may have led to a slight underestimation of mixed type B and C populations. However, in a different study, we genotyped nine microsatellite markers in 345 individuals from 14 populations classified as “type B” here and did not detect any evidence of genetic substructure within samples (our own unpublished data). This result strongly suggests that these samples indeed contain only type B individuals.

If at least one of the four initially genotyped individuals was type C, SNP 3 was genotyped in all remaining individuals sampled from this population. Taking sequencing and pyrosequencing data together, a total of 1337 individuals from 77 populations (62 Swiss, five French and ten German) were analyzed.

### Compilation of data from the literature

To comprehensively understand the distribution of *G. fossarum* species in Central Europe, we combined our results with data from 6 previous publications [15], [16], [17], [18], [32], [47]. Most of these studies ([15], [16], [18], [32], [47]) used allozyme data, which are sufficient to clearly identify type A, but not to distinguish between types B and C. Only one study [17] provides 16S sequencing data that allow the distinction between all three species. The study by Siegismund and Müller [47] does not contain direct information about the *G. fossarum* species. However, from their genetic data it is clear that all individuals belong to the same species, which Siegismund and Müller [47] assume to be *G. fossarum* sensu stricto, i.e. type A.

## Supporting Information

Table S1 ***G. fossarum*** **populations sampled for this study.** Locations are indicated using World Geodetic System (WGS 84) coordinates. The last three columns give the number of individuals analyzed belonging to the three different cryptic species. Bold letters indicate populations where types A and B coexist.
(DOC)

Table S2 **Samples from previous publications shown in** **Figure 1** **.** For each site, name, species composition, drainage and analysis performed to identify species are indicated. With allozyme analysis alone, a distinction between type B and type C is not possible, and species is denoted as “B/C”. The last column indicates the publication which first described species identity. In case a second publication allowed for a refinement of species identification (distinction between types B and C), this one is also indicated.
(DOC)

Table S3 **SNPs analyzed by pyrosequencing for the distinction between** ***G. fossarum*** **types A, B and C.** PCR and pyrosequencing primers are shown. In the sequence analyzed, the diagnostic SNPs are indicated in bold, the first letter corresponding to the *G. fossarum* type(s) first in the alphabet. The last column gives the nucleotide injection order for the pyrosequencer.
(DOC)

## References

[1] Fraser DJ, Bernatchez L (2001). Adaptive evolutionary conservation: towards a unified concept for defining conservation units.. Molecular Ecology.

[2] Mayr E (1963).

[3] Gómez A, Serra M, Carvalho GR, Lunt DH (2002). Speciation in ancient cryptic species complexes: Evidence from the molecular phylogeny of *Brachionus plicatilis* (Rotifera).. Evolution.

[4] De Leaniz CG, Fleming IA, Einum S, Verspoor E, Jordan WC (2007). A critical review of adaptive genetic variation in Atlantic salmon: implications for conservation.. Biological Reviews.

[5] Williams HC, Ormerod SJ, Bruford MW (2006). Molecular systematics and phylogeography of the cryptic species complex *Baetis rhodani* (Ephemeroptera, Baetidae).. Molecular Phylogenetics and Evolution.

[6] MacNeil C, Dick JTA, Elwood RW (1999). The dynamics of predation on *Gammarus* spp. (Crustacea: Amphipoda).. Biological Reviews of the Cambridge Philosophical Society.

[7] MacNeil C, Dick JTA, Elwood RW (1997). The trophic ecology of freshwater *Gammarus* spp. (Crustacea: Amphipoda): Problems and perspectives concerning the functional feeding group concept.. Biological Reviews of the Cambridge Philosophical Society.

[8] Brauer A (1961). Die Süsswasserfauna Deutschlands in einem Band.

[9] Straškraba M (1962). Amphipoden der Tschechoslowakei nach den Sammlungen von Prof.. Hrabě, I. Acta Soc Zool Bohemoslov.

[10] Meijering MPD (1972). Physiologische Beitraege zur Frage der systematischen Stellung von *Gammarus pulex* (L.) und *Gammarus fossarum* KOCH (Amphipoda).. Crustaceana.

[11] Karaman GS, Pinkster S (1977). Freshwater *Gammarus* species from Europe, North Africa and adjacent regions of Asia (Crustacea-Amphipoda). Part I. *Gammarus pulex*-group and related species.. Bijdragen tot de Dierkunde.

[12] Meijering MPD, Hagemann AGL, Schröer HEF (1974). Der Einfluß häuslicher Abwässer auf die Verbreitung von *Gammarus pulex* L. & *Gammarus fossarum* KOCH in einem hessischen Mittelgebirgsbach.. Limnologica.

[13] Meijering MPD (1991). Lack of oxygen and low pH as limiting factors for *Gammarus* in Hessian brooks and rivers.. Hydrobiologia.

[14] Stucki P (2010). Methoden zur Untersuchung und Beurteilung der Fliessgewässer. Makrozoobenthos Stufe F.

[15] Scheepmaker M, van Dalfsen J (1989). Genetic differentiation in *Gammarus fossarum* and *G. carpati* (Crustacea, Amphipoda) with reference to *G. pulex pulex* in northwestern Europe.. Bijdragen tot de Dierkunde.

[16] Müller J (1998). Genetic population structure of two cryptic *Gammarus fossarum* types across a contact zone.. Journal of Evolutionary Biology.

[17] Müller J (2000). Mitochondrial DNA variation and the evolutionary history of cryptic *Gammarus fossarum* types.. Molecular Phylogenetics and Evolution.

[18] Müller J, Partsch E, Link A (2000). Differentiation in morphology and habitat partitioning of genetically characterized *Gammarus fossarum* forms (Amphipoda) across a contact zone.. Biological Journal of the Linnean Society.

[19] Janetzky W (1994). Distribution of the genus *Gammarus* (Amphipoda: Gammaridae) in the River Hunte and its tributaries (Lower Saxony, northern Germany).. Hydrobiologia.

[20] Peeters E, Gardeniers JJP (1998). Logistic regression as a tool for defining habitat requirements of two common gammarids.. Freshwater Biology.

[21] Timm T (1994). *Gammarus fossarum* - ein vergessener Bachflohkrebs im Nordwestdeutschen Tiefland..

[22] Nesemann H, Pöckl M, Wittmann KJ (1995). Distribution of epigean Malacostraca in the middle and upper Danube (Hungary, Austria, Germany).. Miscellanea zoologica hungarica.

[23] Kuhn K, Streit B (1994). Detecting sublethal effects of organophosphates by measuring acetylcholinesterase activity in *Gammarus*.. Bulletin of environmental contamination and toxicology.

[24] Lukančič S, Žibrat U, Mezek T, Jerebic A, Sim i T (2010). Effects of exposing two non-target crustacean species, *Asellus aquaticus* L., and *Gammarus fossarum* Koch., to atrazine and imidacloprid.. Bulletin of Environmental Contamination and Toxicology.

[25] Müller J, Partsch E, Link A, Seitz A (1998). Differentiation of two cryptic *Gammarus fossarum* types in a contact area: morphology, habitat preference, and genetics.. Proceedings of the fourth international crustaceans congress.

[26] Stürzbecher C, Müller J, Seitz A (1998). Coexisting *Gammarus fossarum* types (Amphipoda) in Central Europe: regular patterns of population dynamics and microdistribution.. Proceedings of the Fourth International Crustacean Congress.

[27] Gruber JD, Colligan PB, Wolford JK (2002). Estimation of single nucleotide polymorphism allele frequency in DNA pools by using Pyrosequencing.. Human genetics.

[28] Pinkster S, Scheepmaker M (1994). Hybridization experiments and the taxonomy of *Gammarus* (Amphipoda): A contribution to the understanding of controversial results.. Crustaceana.

[29] Durand JD, Persat H, Bouvet Y (1999). Phylogeography and postglacial dispersion of the chub (*Leuciscus cephalus*) in Europe.. Molecular Ecology.

[30] Kotlik P, Berrebi P (2001). Phylogeography of the barbel (*Barbus barbus*) assessed by mitochondrial DNA variation.. Molecular Ecology.

[31] Hewitt G (2000). The genetic legacy of the Quaternary ice ages.. Nature.

[32] Scheepmaker M (1990). Genetic differentiation and estimated levels of gene flow in members of the *Gammarus pulex*-group (Crustacea, Amphipoda) in western Europe.. Bijdr Dierk.

[33] Stewart JR, Lister AM (2001). Cryptic northern refugia and the origins of the modern biota.. Trends in Ecology & Evolution.

[34] Verovnik R, Sket B, Trontelj P (2005). The colonization of Europe by the freshwater crustacean *Asellus aquaticus* (Crustacea: Isopoda) proceeded from ancient refugia and was directed by habitat connectivity.. Molecular Ecology.

[35] Vonlanthen P, Excoffier L, Bittner D, Persat H, Neuenschwander S (2007). Genetic analysis of potential postglacial watershed crossings in Central Europe by the bullhead (*Cottus gobio* L.).. Molecular Ecology.

[36] Behrmann-Godel J, Gerlach G, Eckmann R (2004). Postglacial colonization shows evidence for sympatric population splitting of Eurasian perch (*Perca fluviatilis* L.) in Lake Constance.. Molecular Ecology.

[37] Barluenga M, Sanetra M, Meyer A (2006). Genetic admixture of burbot (Teleostei: *Lota lota*) in Lake Constance from two European glacial refugia.. Molecular ecology.

[38] Swanson GA (1984). Dissemination of amphipods by waterfowl.. Journal of Wildlife Management.

[39] Strange CD, Glass GB (1979). The distribution of freshwater gammarids in Northern Ireland.. Proceedings of the Royal Irish Academy.

[40] Hardin G (1960). The competitive exclusion principle.. Science.

[41] Dick JTA, Montgomery I, Elwood RW (1993). Replacement of the indigenous amphipod *Gammarus duebeni* celticus by the introduced *G. pulex*: differential cannibalism and mutual predation.. Journal of Animal Ecology.

[42] Brown WL, Wilson EO (1956). Character displacement.. Systematic Zoology.

[43] Eggers TO, Martens A (2001). Bestimmungsschlüssel der Süsswasser-Amphipoda (Crustacea) Deutschlands. A key to the freshwater Amphipods (Crustacea) of Germany.. Lauterbornia.

[44] Kinzelbach R, Claus W (1977). Die Verbreitung von *Gammarus fossarum* Koch, 1835, *G. pulex* (Linnaeus, 1758) und *G. roeselii* Gervais, 1835, in den linken Nebenflüssen des Rheins zwischen Wieslauter und Nahe.. Crustaceana Supplement.

[45] Montero-Pau J, Gómez A, Muñoz J (2008). Application of an inexpensive and high-throughput genomic DNA extraction method for the molecular ecology of zooplanktonic diapausing eggs.. Limnology and Oceanography: Methods.

[46] Ronaghi M, Uhlen M, Nyren P (1998). A sequencing method based on real-time pyrophosphate.. Science.

[47] Siegismund HR, Müller J (1991). Genetic structure of *Gammarus fossarum* populations.. Heredity.

