# Supplementary material for: Spatial Distribution of Cryptic Species Diversity in European Freshwater Amphipods (Gammarus fossarum) as Revealed by Pyrosequencing
Source: PLoS One. 2011 Aug 31;6(8):e23879. doi: 10.1371/journal.pone.0023879 (PMC3166083; doi:10.1371/journal.pone.0023879)
Supplement: Table S1 — G. fossarum populations sampled for this study. Locations are indicated using World Geodetic System (WGS 84) coordinates. The last three columns give the number of individuals analyzed belonging to the three different cryptic species. Bold letters indicate populations where types A and B coexist. (DOC) [file pone.0023879.s001.doc]

**Table S1.** ***G. fossarum* populations sampled for this study.** Locations are indicated using World Geodetic System (WGS 84) coordinates. The last three columns give the number of individuals analyzed belonging to the three different cryptic species. Bold letters indicate populations where types A and B coexist.

| **abbre-viation** | **country** | **sampling date** | **latitude** | **longi-tude** | **drai-nage** | **A** | **B** | **C** |
| --- | --- | --- | --- | --- | --- | --- | --- | --- |
| BEF | France | 16./17.2.2010 | 47.377 | 6.598 | Rhone | 0 | 0 | 10 |
| COF | France | 16./17.2.2010 | 45.250 | 5.766 | Rhone | 0 | 9 | 0 |
| LC | France | 11.11.2009 | 46.263 | 6.028 | Rhone | 0 | 31 | 0 |
| SLF | France | 16./17.2.2010 | 45.357 | 5.756 | Rhone | 0 | 2 | 0 |
| SOF | France | 16./17.2.2010 | 47.265 | 4.587 | Rhone | 0 | 9 | 0 |
| AN | Germany | ? | 47.809 | 8.315 | Rhine | 19 | 0 | 0 |
| MS01 | Germany | 18.08.2008 | 51.532 | 8.531 | Rhine | 0 | 1 | 0 |
| MS02 | Germany | 18.08.2008 | 51.524 | 8.695 | Rhine | 0 | 1 | 0 |
| MS03 | Germany | 18.08.2008 | 51.502 | 8.719 | Rhine | 0 | 1 | 0 |
| MS04 | Germany | 18.08.2008 | 51.582 | 8.580 | Rhine | 0 | 1 | 0 |
| MS05 | Germany | 19.08.2008 | 51.646 | 8.735 | Rhine | 0 | 1 | 0 |
| MS06 | Germany | 20.08.2008 | 51.593 | 8.832 | Rhine | 0 | 1 | 0 |
| MS08 | Germany | 20.08.2008 | 51.543 | 8.909 | Rhine | 0 | 1 | 0 |
| MS09 | Germany | 20.08.2008 | 51.663 | 8.897 | Rhine | 0 | 1 | 0 |
| MS10 | Germany | 20.08.2008 | 51.721 | 8.717 | Rhine | 0 | 1 | 0 |
| SP | Switzerland | ? | 46.651 | 10.124 | Danube | 19 | 0 | 0 |
| PC | Switzerland | 24.09.2008 | 46.297 | 10.083 | Po | 7 | 0 | 0 |
| AA | Switzerland | 05.06.2008 | 47.350 | 8.694 | Rhine | 13 | 0 | 0 |
| AL | Switzerland | 15.03.2009 | 47.121 | 7.334 | Rhine | 0 | 15 | 0 |
| AM | Switzerland | 24.04.2008 | 47.280 | 8.382 | Rhine | 0 | 42 | 0 |
| B | Switzerland | 02.04.2009 | 46.755 | 7.318 | Rhine | 13 | 0 | 0 |
| BI | Switzerland | 10.04.2008 | 47.122 | 8.349 | Rhine | 4 | 0 | 0 |
| BN | Switzerland | 08.10.2009 | 47.148 | 9.035 | Rhine | 0 | 19 | 0 |
| BS | Switzerland | 10.06.2008 | 47.399 | 8.595 | Rhine | 12 | 0 | 0 |
| BW | Switzerland | 13.08.2008 | 47.316 | 8.184 | Rhine | 15 | 0 | 0 |
| **CN** | **Switzerland** | **16.03.2009** | **47.347** | **7.481** | **Rhine** | **8** | **16** | **0** |
| CO | Switzerland | 23.11.2007 | 46.930 | 6.629 | Rhine | 0 | 0 | 4 |
| CS | Switzerland | 21.09.2008 | 47.444 | 9.370 | Rhine | 24 | 0 | 0 |
| DB | Switzerland | 10.04.2008 | 47.180 | 8.415 | Rhine | 0 | 40 | 0 |
| DI | Switzerland | 20.08.2008 | 46.842 | 7.645 | Rhine | 3 | 0 | 0 |
| EM | Switzerland | 09.12.2007 | 46.957 | 7.389 | Rhine | 0 | 25 | 0 |
| F | Switzerland | 02.04.2009 | 46.892 | 7.304 | Rhine | 0 | 24 | 0 |
| FB | Switzerland | 05.06.2008 | 47.246 | 8.785 | Rhine | 17 | 0 | 0 |
| FL | Switzerland | 01.05.2008 | 46.851 | 6.510 | Rhine | 0 | 0 | 3 |
| **G** | **Switzerland** | **15.10.2009** | **46.794** | **7.401** | **Rhine** | **10** | **12** | **0** |
| **G4** | **Switzerland** | **23.07.2009** | **47.337** | **7.279** | **Rhine** | **20** | **37** | **0** |
| GB | Switzerland | 25.11.2007 | 46.711 | 7.628 | Rhine | 0 | 7 | 0 |
| GH | Switzerland | 28.04.2009 | 47.591 | 8.744 | Rhine | 2 | 0 | 0 |
| **GL** | **Switzerland** | **01.05.2008** | **47.332** | **7.223** | **Rhine** | **2** | **42** | **0** |
| GR | Switzerland | 01.05.2008 | 46.755 | 6.639 | Rhine | 0 | 23 | 0 |
| HM | Switzerland | 26.11.2007 | 47.078 | 7.751 | Rhine | 4 | 0 | 0 |
| HU | Switzerland | 24.02.2009 | 47.581 | 8.672 | Rhine | 18 | 0 | 0 |
| HW | Switzerland | 26.11.2007 | 47.123 | 7.908 | Rhine | 0 | 5 | 0 |
| JA | Switzerland | 20.08.2008 | 46.850 | 7.691 | Rhine | 29 | 0 | 0 |
| KA | Switzerland | 25.11.2007 | 46.692 | 7.632 | Rhine | 7 | 0 | 0 |
| KB | Switzerland | 26.03.2008 | 47.002 | 8.333 | Rhine | 0 | 5 | 0 |
| KD | Switzerland | ? | 47.319 | 8.609 | Rhine | 24 | 0 | 0 |
| **KE** | **Switzerland** | **26.11.2007** | **47.054** | **8.090** | **Rhine** | **1** | **3** | **0** |
| KL | Switzerland | 13.08.2008 | 47.282 | 8.057 | Rhine | 0 | 44 | 0 |
| **LG** | **Switzerland** | **29.04.2008** | **46.603** | **6.953** | **Rhine** | **3** | **27** | **0** |
| **MR** | **Switzerland** | **23.05.2008** | **47.459** | **8.244** | **Rhine** | **3** | **12** | **0** |
| MW | Switzerland | 10.04.2008 | 47.234 | 8.423 | Rhine | 0 | 39 | 0 |
| NT | Switzerland | 08.10.2009 | 47.077 | 9.053 | Rhine | 19 | 0 | 0 |
| PE | Switzerland | 10.04.2008 | 47.111 | 8.362 | Rhine | 0 | 34 | 0 |
| RB | Switzerland | 26.11.2007 | 47.029 | 7.682 | Rhine | 4 | 0 | 0 |
| RM | Switzerland | 13.08.2008 | 47.249 | 7.989 | Rhine | 0 | 33 | 0 |
| RO | Switzerland | 25.11.2007 | 46.810 | 7.589 | Rhine | 4 | 0 | 0 |
| **RS** | **Switzerland** | **24.04.2008** | **47.319** | **8.374** | **Rhine** | **1** | **30** | **0** |
| RT | Switzerland | 15.03.2009 | 47.158 | 7.413 | Rhine | 0 | 35 | 0 |
| **RW** | **Switzerland** | **19.08.2008** | **47.076** | **8.109** | **Rhine** | **17** | **2** | **0** |
| SB | Switzerland | 23.05.2008 | 47.476 | 8.211 | Rhine | 0 | 30 | 0 |
| SC | Switzerland | 23.05.2008 | 47.487 | 8.217 | Rhine | 0 | 8 | 0 |
| TT | Switzerland | 25.03.2008 | 47.428 | 8.838 | Rhine | 21 | 0 | 0 |
| TU | Switzerland | 21.09.2008 | 47.487 | 9.452 | Rhine | 17 | 0 | 0 |
| UE | Switzerland | 18.07.2007 | 47.578 | 8.835 | Rhine | 18 | 0 | 0 |
| UT | Switzerland | 20.08.2008 | 46.794 | 7.573 | Rhine | 0 | 24 | 0 |
| VI | Switzerland | 18.05.2009 | 47.163 | 7.032 | Rhine | 0 | 34 | 0 |
| WB | Switzerland | 05.06.2008 | 47.306 | 8.824 | Rhine | 7 | 0 | 0 |
| WT | Switzerland | 25.03.2008 | 47.480 | 8.708 | Rhine | 9 | 0 | 0 |
| WY | Switzerland | 23.05.2008 | 47.264 | 7.827 | Rhine | 0 | 4 | 0 |
| AP | Switzerland | 30.04.2008 | 46.212 | 7.315 | Rhone | 0 | 46 | 0 |
| CP | Switzerland | 11.11.2009 | 46.325 | 6.173 | Rhone | 0 | 28 | 0 |
| DU | Switzerland | 11.11.2009 | 46.427 | 6.294 | Rhone | 0 | 31 | 0 |
| LM | Switzerland | 01.05.2008 | 47.302 | 7.059 | Rhone | 0 | 0 | 9 |
| PF | Switzerland | 30.04.2008 | 46.298 | 7.596 | Rhone | 0 | 33 | 0 |
| PN | Switzerland | 19.05.2009 | 47.421 | 7.078 | Rhone | 0 | 40 | 0 |
| SM | Switzerland | 30.04.2008 | 46.207 | 7.009 | Rhone | 0 | 8 | 0 |
