# Supplementary material for: Spatial Distribution of Cryptic Species Diversity in European Freshwater Amphipods (Gammarus fossarum) as Revealed by Pyrosequencing
Source: PLoS One. 2011 Aug 31;6(8):e23879. doi: 10.1371/journal.pone.0023879 (PMC3166083; doi:10.1371/journal.pone.0023879)
Supplement: Table S2 — Samples from previous publications shown in Figure 1 . For each site, name, species composition, drainage and analysis performed to identify species are indicated. With allozyme analysis alone, a distinction between type B and type C is not possible, and species is denoted as “B/C”. The last column indicates the publication which first described species identity. In case a second publication allowed for a refinement of species identification (distinction between types B and C), this one is also indicated. (DOC) [file pone.0023879.s002.doc]

**Table S2. Samples from previous publications shown in Figure 1.** For each site, name, species composition, drainage and analysis performed to identify species are indicated. With allozyme analysis alone, a distinction between type B and type C is not possible, and species is denoted as "B/C". The last column indicates the publication which first described species identity. In case a second publication allowed for a refinement of species identification (distinction between types B and C), this one is also indicated.

| **site name** | **species** | **drainage** | **analysis type** | **reference** |
| --- | --- | --- | --- | --- |
| 18 | B/C | Arc | allozyme | [1] |
| 1 | A | Danube | allozyme | [2] |
| 2 | A | Danube | allozyme | [2] |
| 3 | A | Danube | allozyme | [2] |
| Alb | A | Danube | allozyme | [3] |
| Alt | A | Danube | allozyme | [3] |
| Apf | A | Danube | allozyme | [3] |
| Att | A | Danube | allozyme | [3] |
| Bau | A | Danube | allozyme | [3] |
| Bur | A | Danube | allozyme | [3] |
| Doc | A | Danube | allozyme | [3] |
| Doe | A | Danube | allozyme | [3] |
| Don | A | Danube | allozyme | [3] |
| Ffb | A | Danube | allozyme | [3] |
| Gra | A | Danube | allozyme | [3] |
| Gro | A | Danube | allozyme | [3] |
| Hil | A | Danube | allozyme | [3] |
| Hor | A | Danube | allozyme | [3] |
| Ink | A | Danube | allozyme | [3] |
| Kar | A | Danube | allozyme | [3] |
| Koe | A | Danube | allozyme | [3] |
| Lau | A | Danube | allozyme | [3] |
| Len | A | Danube | allozyme | [3] |
| Loi | A | Danube | allozyme | [3] |
| Lon | A | Danube | allozyme | [3] |
| Mar | A | Danube | allozyme | [3] |
| Mer | A | Danube | allozyme | [3] |
| Min | A | Danube | allozyme | [3] |
| Rai | A | Danube | allozyme | [3] |
| REI | A | Danube | allozyme | [4] |
| San | A | Danube | allozyme | [3] |
| Sch | A | Danube | allozyme | [3] |
| See | A | Danube | allozyme | [3] |
| Ste | A | Danube | allozyme | [3] |
| Sto | A | Danube | allozyme | [3] |
| Tue | A | Danube | allozyme | [3] |
| Ulm | A | Danube | allozyme | [3] |
| Vor | A | Danube | allozyme | [3] |
| Wel | A | Danube | allozyme | [3] |
| 11 | B/C | Liane | allozyme | [2] |
| 7 | B/C | Meuse | allozyme | [2] |
| 8/SAM | B | Meuse | allozyme, 16S sequencing | [2]; [5] |
| LOM | B | Meuse | allozyme, 16S sequencing | [4]; [5] |
| SOM | mix B and C | Meuse | allozyme, 16S sequencing | [4]; [5] |
| 4 | B/C | Rhine | allozyme | [2] |
| 6 | B/C | Rhine | allozyme | [2] |
| Ams | A | Rhine | allozyme | [3] |
| AUL1 | A | Rhine | allozyme | [6] |
| AUL2 | mix A and B/C | Rhine | allozyme | [6] |
| BON | A | Rhine | allozyme | [6] |
| COL | mix A and B/C | Rhine | allozyme | [6] |
| DAC | B/C | Rhine | allozyme | [6] |
| DAH | mix A and B/C | Rhine | allozyme | [4] |
| DIE | mix A and B/C | Rhine | allozyme | [6] |
| DOE | mix A and B | Rhine | allozyme, 16S sequencing | [4]; [5] |
| DOR | mix A and B | Rhine | allozyme, 16S sequencing | [4]; [5] |
| EIC | mix A and B/C | Rhine | allozyme | [6] |
| EMM | A | Rhine | allozyme | [6] |
| ENZ | B | Rhine | allozyme, 16S sequencing | [4]; [5] |
| ETT | mix A and B/C | Rhine | allozyme | [6] |
| Eya | A | Rhine | allozyme | [3] |
| FRI | A | Rhine | allozyme, 16S sequencing | [5] |
| GAI | B | Rhine | allozyme, 16S sequencing | [4]; [5] |
| GAU | B | Rhine | allozyme, 16S sequencing | [4]; [5] |
| HAI1 | B/C | Rhine | allozyme | [6] |
| HAI2 | B/C | Rhine | allozyme | [6] |
| HAP | B/C | Rhine | allozyme | [6] |
| HAU | B/C | Rhine | allozyme | [6] |
| HEI | A | Rhine | allozyme | [4] |
| HOH | A | Rhine | allozyme | [6] |
| HOM | B | Rhine | allozyme, 16S sequencing | [4]; [5] |
| HUB | B | Rhine | allozyme, 16S sequencing | [4]; [5] |
| IPH | A | Rhine | allozyme | [4] |
| KAP | B | Rhine | allozyme, 16S sequencing | [4]; [5] |
| KIT | B/C | Rhine | allozyme | [6] |
| KLE | B | Rhine | allozyme, 16S sequencing | [5] |
| LAH | B/C | Rhine | allozyme | [6] |
| LIG | A | Rhine | allozyme | [6] |
| LOC | A | Rhine | allozyme | [6] |
| LOI | A | Rhine | allozyme, 16S sequencing | [5] |
| LUX | C | Rhine | allozyme, 16S sequencing | [4]; [5] |
| MUN | mix A and B | Rhine | allozyme, 16S sequencing | [5] |
| NEU | A | Rhine | allozyme | [4] |
| OBE | B | Rhine | allozyme, 16S sequencing | [5] |
| OLS | A | Rhine | allozyme | [4] |
| OST | A | Rhine | allozyme | [6] |
| Seb | A | Rhine | allozyme | [3] |
| SEE | mix A and B/C | Rhine | allozyme | [6] |
| SEM | mix A and B | Rhine | allozyme, 16S sequencing | [5] |
| SIG | A | Rhine | allozyme | [6] |
| SIL | B/C | Rhine | allozyme | [6] |
| STE | B/C | Rhine | allozyme | [6] |
| STI | B/C | Rhine | allozyme | [6] |
| STJ | B | Rhine | allozyme, 16S sequencing | [4]; [5] |
| STO | A | Rhine | allozyme | [6] |
| SUL | A | Rhine | allozyme | [6] |
| TEN | A | Rhine | allozyme | [6] |
| TOD | A | Rhine | allozyme | [6] |
| WAE | B | Rhine | allozyme, 16S sequencing | [4]; [5] |
| WAL | A | Rhine | allozyme | [6] |
| Wie | A | Rhine | allozyme | [3] |
| WOL | mix A and B | Rhine | allozyme, 16S sequencing | [4]; [5] |
| ZIM | A | Rhine | allozyme | [6] |
| ZWE | B | Rhine | allozyme, 16S sequencing | [4]; [5] |
| 9 | B/C | Rhone | allozyme | [1] |
| 10 | B/C | Rhone | allozyme | [1] |
| 11 | B/C | Rhone | allozyme | [1] |
| 12 | B/C | Rhone | allozyme | [1] |
| 13 | B/C | Rhone | allozyme | [1] |
| 14 | B/C | Rhone | allozyme | [1] |
| 15 | B/C | Rhone | allozyme | [1] |
| 16 | B/C | Rhone | allozyme | [1] |
| DEL | mix A and B/C | Rhone | allozyme | [6] |
| 13 | B/C | Slack | allozyme | [2] |
| 14 | B/C | Slack | allozyme | [2] |
| NWH | B | Weser | allozyme, 16S sequencing | [4]; [5] |
| 12 | B/C | Wimereux | allozyme | [2] |

**References**

**1. Scheepmaker M (1990) Genetic differentiation and estimated levels of gene flow in members of the *Gammarus pulex*-group (Crustacea, Amphipoda) in western Europe. Bijdr Dierk 60: 3-30.**

**2. Scheepmaker M, van Dalfsen J (1989) Genetic differentiation in *Gammarus fossarum* and *G. carpati* (Crustacea, Amphipoda) with reference to *G. pulex pulex* in northwestern Europe. Bijdragen tot de Dierkunde 59: 127-139.**

**3. Siegismund HR, Müller J (1991) Genetic structure of *Gammarus fossarum* populations. Heredity 66: 419-436.**

**4. Müller J (1998) Genetic population structure of two cryptic *Gammarus fossarum* types across a contact zone. Journal of Evolutionary Biology 11: 79-101.**

**5. Müller J (2000) Mitochondrial DNA variation and the evolutionary history of cryptic *Gammarus fossarum* types. Molecular Phylogenetics and Evolution 15: 260-268.**

**6. Müller J, Partsch E, Link A (2000) Differentiation in morphology and habitat partitioning of genetically characterized *Gammarus fossarum* forms (Amphipoda) across a contact zone. Biological Journal of the Linnean Society 69: 41-53.**
