# Supplementary material for: Spatial Distribution of Cryptic Species Diversity in European Freshwater Amphipods (Gammarus fossarum) as Revealed by Pyrosequencing
Source: PLoS One. 2011 Aug 31;6(8):e23879. doi: 10.1371/journal.pone.0023879 (PMC3166083; doi:10.1371/journal.pone.0023879)
Supplement: Table S3 — SNPs analyzed by pyrosequencing for the distinction between G. fossarum types A, B and C. PCR and pyrosequencing primers are shown. In the sequence analyzed, the diagnostic SNPs are indicated in bold, the first letter corresponding to the G. fossarum type(s) first in the alphabet. The last column gives the nucleotide injection order for the pyrosequencer. (DOC) [file pone.0023879.s003.doc]

**Table S3. SNPs analyzed by pyrosequencing for the distinction between *G. fossarum* types A, B and C.** PCR and pyrosequencing primers are shown. In the sequence analyzed, the diagnostic SNPs are indicated in bold, the first letter corresponding to the *G. fossarum* type(s) first in the alphabet. The last column gives the nucleotide injection order for the pyrosequencer.

| **SNP ID** | **distinction** | **primers (5'-3')** | **sequence analyzed (diagnostic SNPs in bold)** | **nucleotide injection order** |
| --- | --- | --- | --- | --- |
| 1 & 2 | A vs. B/C | PCR forward: biotin-TTAACCYTTCATTCMAGTTTTCAA | GC**T/G**TAATCA**G/A** | TGCGTGATCGAG |
|  |  | PCR reverse: AAGTAAAACCTGCCCGGTGCTT (LR-N-Gf in Müller (2000)) |  |  |
|  |  | pyrosequencing: TGACTGTGCTAAGGTA |  |  |
| 3 | A/B vs. C | PCR forward: biotin-AATACCGCACTGTTATCCCTA | ATTRGT**G/A**TTTTAA | GATAGCTAGCTA |
|  |  | PCR reverse: GGGACGATAAGACCCTAGAAG |  |  |
|  |  | pyrosequencing: TTTTTACTGGGGCGGT |  |  |
